# Modeling Tool for Studying the Influence of Operating Conditions on the Enzymatic Hydrolysis of Milk Proteins

**DOI:** 10.3390/foods11244080

**Published:** 2022-12-16

**Authors:** Pedro Valencia, Karen Espinoza, Carolina Astudillo-Castro, Fernando Salazar

**Affiliations:** 1 Department of Chemical and Environmental Engineering, Universidad Técnica Federico Santa María, Avenida España 1680, Valparaíso 2390123, Chile; 2 School of Food Engineering, Pontificia Universidad Católica de Valparaíso, Waddington 716, Valparaíso 2360100, Chile

**Keywords:** milk protein, protein hydrolysis, enzymatic hydrolysis, mathematical modeling

## Abstract

Systematic modeling of the enzymatic hydrolysis of milk proteins is needed to assist the study and production of partially hydrolyzed milk. The enzymatic hydrolysis of milk proteins was characterized and evaluated as a function of the temperature and protease concentration using Alcalase, Neutrase and Protamex. Modeling was based on the combination of two empirical models formed by a logarithmic and a polynomial equation to correlate the kinetic constants and the operating conditions. The logarithmic equation fitted with high accuracy to the experimental hydrolysis curves with the three proteases (*R*^2^ > 0.99). The kinetic constants were correlated with the operating conditions (*R*^2^ > 0.97) using polynomial equations. The temperature and protease concentration significantly affected the initial rate of hydrolysis, i.e., the kinetic constant *a*, while the kinetic constant *b* was not significantly affected. The values for the kinetic constant *a* were predicted according to the operating conditions and they were strongly correlated with the experimental data (*R*^2^ = 0.95). The model allowed for a high-quality prediction of the hydrolysis curves of milk proteins. This modeling tool can be used in future research to test the correlation between the degree of hydrolysis and the functional properties of milk hydrolysates.

## 1. Introduction

The use of hydrolyzed milk proteins is a very attractive practice directed to children’s food not only for the hypoallergenicity of this product, but also for the immunomodulatory action of enzyme-generated peptides [1]. The hydrolyzed proteins are obtained by the proteolytic action of proteases, which reduces the molecular weight of proteins hydrolyzing the peptide bonds, thus decreasing the allergenicity caused by epitopes [2,3]. An increase in the degree of hydrolysis and the levels of low molecular weight peptides are generally correlated with the reduced antigenicity of milk proteins [4]. Wroblewska, et al. [5] found that an increase in the degree of hydrolysis (DH) from 15.3% to 15.9% after a two-step process with Alcalase and papain showed a more effective decrease in immunoreactivity despite the nonsignificant increase in the DH. This is a key control parameter during the production of partially hydrolyzed formulas, which could be preferred over extensively hydrolyzed formulas because of their better taste and nutritional value [2]. Partially hydrolyzed formulas are more orally tolerated and less expensive than extensively hydrolyzed formulas [6]. The DH is a useful parameter to characterize the progression of the proteolytic reaction and it is influenced by the operating conditions, traditionally pH, temperature, protease dose and protease-to-substrate ratio [7]. As mentioned above, control of the DH is a major concern during the formulation of hypoallergenic milk. The correlation of DH with hypoallergenicity is a common strategy to determine the adequate DH to be achieved. In addition, the DH achieved depends on the operating conditions and the protease used. The characterization of the hydrolysis process regarding the operating conditions must be evaluated before allergenicity tests. Theoretical modeling by the Michaelis-Menten equation has been proposed by various authors with different protein substrates and proteases [8,9,10,11,12,13,14,15]. The logarithmic equation obtained by Márquez-Moreno and Fernández-Cuadrado [16] has been used by different authors [17,18,19,20,21,22,23,24] due to its simplicity and successful fitting to the experimental data. Empirical modeling based on response surface methodology (RSM) has been applied to protein hydrolysis [25,26,27,28,29]. In a recent publication, a two-level structured model was used as a new methodology to characterize the enzymatic hydrolysis of proteins [30]. The advantages of this methodology are (i) a good description of the hydrolysis process, (ii) an operating definition of kinetic constants, (iii) excellent fitting to experimental data, (iv) the simultaneous exploration of multiple variables (operating conditions) and (v) very good predictability.

The objective of this work was to characterize the enzymatic hydrolysis of milk proteins regarding the operating conditions with different proteases using this recently published modeling methodology. The proposed methodology can be used to correlate the DH with immunoreactivity reduction during the hydrolysis of milk proteins. The information obtained will be useful to produce hypoallergenic milk by designing and setting the operating conditions, thus achieving the proper DH during the process.

## 2. Materials and Methods

### 2.1. Materials

Milk powder from Hormel Foods USA was used. The milk contained 33.7% (*w*/*w*) protein and 5.3% (*w*/*w*) moisture. Commercial proteases Alcalase 2.4 L (serine endoprotease from *Bacillus licheniformis*), Neutrase (metallo-endoprotease from *Bacillus amyloliquefaciens*) and Protamex (a protease with endoprotease and exopeptidase activity from *Bacillus licheniformis* and *Bacillus amyloliquefaciens*) supplied by Novozymes (Bagsvaerd, Denmark) were used. Analytical grade quality reagents were used in all experiments.

### 2.2. Hydrolysis Curves

The milk was prepared by mixing the low-heat milk powder with deionized water at a 1:10 proportion, resulting in a 3.1% (*w*/*w*) protein solution. The mixture was subjected to magnetic agitation and the homogenized mixture is hereafter delineated milk. The milk was loaded in a glass vessel and thermoregulated by a water bath at a constant temperature. After thermal equilibrium, the pH was fixed at 6.5. The protease previously diluted with deionized water was added to start the hydrolysis reaction. Different protease amounts were added to 40 g of milk. The release of free α-amino groups was followed by the pH-stat technique (G20 Compact Titrator, Mettler-Toledo) to obtain the hydrolysis progress. The pH was constantly monitored and 0.5 N NaOH was added to maintain the pH of 6.5 during the 60 min of reaction. Calculations were performed according to Equation (1):

(1) $$\alpha \mathrm{-NH}\;\left(\mathrm{mM}\right)=\frac{V\cdot N}{\alpha \cdot V_{T}}$$

In the above equation, *V* is the added NaOH volume, *N* is the NaOH concentration, *V_T_* is the total reaction volume and *α* is the average degree of dissociation of the α-NH groups (an average value of 0.2 was used for all conditions). The total α-amino groups in milk proteins were determined by quantification with the *o*-phthaldialdehyde method (OPA) reported by Nielsen [31] after total hydrolysis in 6 N HCl at 110 °C for 24 h. The *DH* in each experiment was calculated according to Equation (2):

(2) $$DH=\frac{V\cdot N}{\alpha \cdot h_{T}\cdot M_{P}}$$

where *h*_T_ is the content of α-amino groups per protein mass in milk (10.7 meq/g_prot_) and *M*_P_ is the mass of protein in the reaction mixture (1.24 g). The release of α-amino groups against time was plotted to build the hydrolysis curves.

### 2.3. Experimental Design and Statistics

A central composite circumscribed (CCC) design was used to set the number and conditions of each hydrolysis experiment. Each experiment corresponded to a hydrolysis curve with fixed reaction conditions of temperature (T) and protease concentration (E). The T and E values resulting from the design were between 48–62 °C and 19–231 mAU, respectively (Table 1). A total of 10 experiments resulted from the CCC design shown in Table 2. Each experiment was randomly run as one replicate. The central point was replicated in Experiments 5 and 10 (Table 2). Each experiment consisted of a hydrolysis curve where the product concentration (α-NH) was registered against the reaction time. The autotitrator registered one data point per second, resulting in 3600 points of product concentration (α-NH) as a function of time for a 1 h experiment.

The first modeling level corresponds to the description of each hydrolysis curve performed by the estimation of the kinetics constants *a* and *b* from Equation (3). The logarithmic equation proposed by Márquez-Moreno and Fernández-Cuadrado [16] in terms of DH can be rewritten in terms of product concentration:

(3) $$P=\frac{1}{b}\ln(abt+1)$$

where *P* is the concentration of released α-amino groups during milk protein hydrolysis, *t* is the reaction time and *a* and *b* are the kinetic constants of the model. These constants were estimated by the nonlinear least-squares method by fitting Equation (3) to the experimental data. The determination coefficient (R^2^) and the standard error (*se*) were used to characterize the fitting quality according to Equation (4):

(4) $$se=\sqrt{{\hat{\sigma }}^{2}\cdot C_{jj}},$$

where ${\hat{\sigma }}^{2}$ is the estimator of *σ*^2^ calculated from the mean sum of squares and *C_jj_* is the diagonal element of the dispersion matrix (*X*^T^*X*)^−1^. The second modeling level correlated each of the kinetic constants *a* and *b* with the reaction conditions. Equation (5) resulted from considering the variables T (*x*_1_) and E (*x*_2_) (operating conditions) and the combination of linear, square and interaction components.

(5) $$y=\beta_{0}+\beta_{1}x_{1}+\beta_{2}x_{2}+\beta_{11}x_{1}^{2}+\beta_{22}x_{2}^{2}+\beta_{12}x_{1}x_{2}$$

A multivariable regression was performed to estimate the regression coefficients (*β_i_*). A *t* test was used to calculate the statistical significance of each individual regression coefficient. This information was used to eliminate the nonsignificant regression coefficients and reduce the polynomial model. An analysis of variance (ANOVA) was used to test the significance of the regression (Table 3, Table 4 and Table 5). Additional hydrolysis experiments were performed to test the predictability of the model (Table 6). The prediction range was calculated from the confidence intervals according to Equation (6):

(6) $$\hat{y}\left(x_{0}\right)\pm t_{\alpha /2,n-p}\sqrt{{\hat{\sigma }}^{2}\cdot x_{0}{}^{T}{\left(X^{T}X\right)}^{-1}x_{0}}$$

where *x*_0_ corresponds to the vector of the experimental points considered in the validation experiment and $x_{0}{}^{T}{\left(X^{T}X\right)}^{-1}x_{0}$ is the prediction variance used to calculate the prediction error of the kinetic constants *a* and *b*.

## 3. Results and Discussion

**Figure 1:**
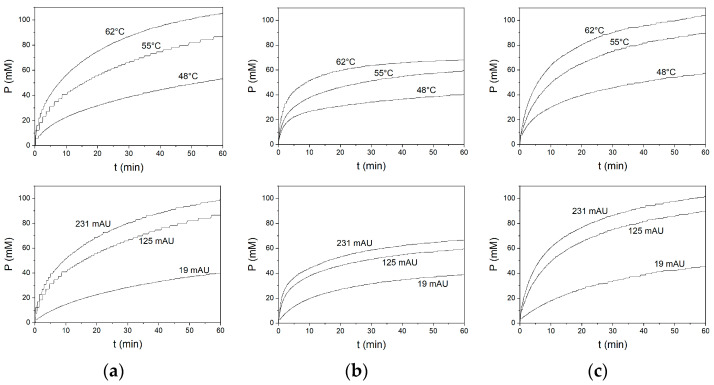
Experimental curves of milk protein hydrolysis with (**a**) Alcalase, (**b**) Neutrase and (**c**) Protamex at different temperatures (125 mAU) and protease concentrations (55 °C).

The description of the enzymatic hydrolysis of milk proteins was assessed through a two-level mathematical model using a previously published methodology [30]. The first-level model corresponds to the logarithmic equation used to model a wide spectrum of hydrolysis curves with different protein sources and proteases. The effect of E and T on the reaction performance can be observed in Figure 1. The hydrolysis curves generated similar results for Alcalase and Protamex. On the other hand, Neutrase exhibited high efficiency at the initial phase of the hydrolysis curve (high *a* values) and a sharp decrease in the hydrolysis rate after 5 or 10 min of reaction. Consequently, lower degrees of hydrolysis were obtained with Neutrase compared to Alcalase and Protamex.

These results agree with previous observations concerning Neutrase thermal stability. Neutrase activity has an optimal temperature range of 45 °C to 55 °C and its activity sharply decreases at temperatures over 55 °C [32]. The half-life was estimated to be approximately 10 min at 60 °C and a pH of 7. This important decrease in Neutrase activity explains the observed shape of the hydrolysis curve. The logarithmic equation was fit to determine the kinetic constants *a* and *b* in each experiment. These results are shown in Table 2 for Alcalase, Neutrase and Protamex. A high fitting quality was obtained for all the hydrolysis curves (*R*^2^ > 0.99). This result agrees with previous fittings of hydrolysis curves [22,30]. As stated previously, the kinetic constant *a* corresponds to the initial slope of the hydrolysis curve, i.e., dP/dt at t=0 [30,33]. Thus, it can be predicted that the kinetic constant *a* increases when T and E increase. It can be observed in Figure 1 that faster hydrolysis rates and larger reaction extensions were obtained at higher temperatures and protease concentrations. The highest values for the kinetic constant *a* and the lowest values for the kinetic constant *b* were obtained with Neutrase. This is reflected in a high initial rate and an abrupt decrease after 5 or 10 min of reaction. Despite this high initial rate, Neutrase produced the lowest DHs. On the other hand, Alcalase and Protamex produced lower initial rates but higher DHs than Neutrase. This result can be explained by the higher thermal stability of Alcalase and Protamex. Valencia et al. [15] reported an Alcalase activity loss of 20% after 3 h of reaction during the hydrolysis of 1.7% (*w*/*w*) salmon muscle proteins.

**Figure 2:**
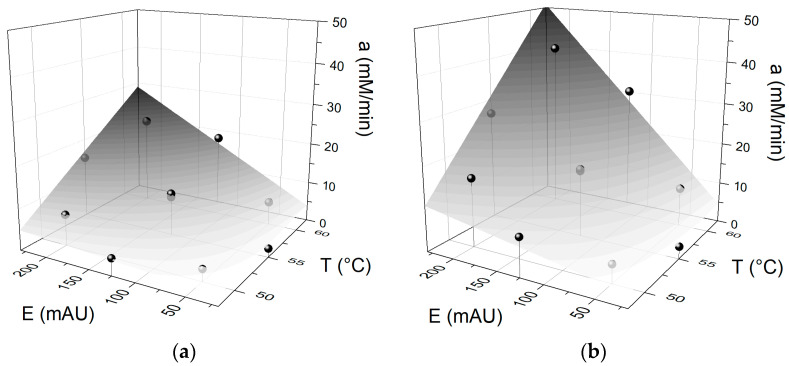
Response surfaces for kinetic constant *a* against the operating conditions T and E for the hydrolysis of milk proteins with (**a**) Alcalase and (**b**) Protamex.

According to the obtained results, the kinetic constants *a* and *b* can characterize the different catalytic efficiencies that proteases exhibit during milk protein hydrolysis. The second modeling level was based on the correlation between the kinetic constants (*a*, *b*) and the reaction conditions T and E. The polynomial equation contained T and E as the independent variables. The multivariable regression results are shown in Table 3, Table 4 and Table 5 for Alcalase, Neutrase and Protamex, respectively. The nonsignificant coefficients (*β*_i_) were eliminated from the polynomial equation to obtain a reduced model for each protease (Table 3, Table 4 and Table 5). The reaction conditions significantly affected the kinetic constant *a* for all proteases. A lower correlation between the kinetic constant *a* and operating conditions was obtained with Neutrase (*R*^2^ = 0.8746) compared to Alcalase and Protamex. This can be explained by the lower thermal stability of Neutrase. A better correlation can be observed at lower temperatures for Neutrase (40–50 °C). The kinetic constant *b* was poorly correlated with the operating conditions when Alcalase and Protamex were used (*R*^2^ = 0.7972 and *R*^2^ = 0.8693, respectively). For this reason, the kinetic constant *b* was fixed at 0.0410 and 0.0423 for Alcalase and Protamex, respectively, corresponding to the central value. The reaction conditions impacted the kinetic constant *b* to a lesser extent than the kinetic constant *a*. Average values of *b* for different operating conditions have been used in previous publications [16,19,21,22,24]. This result is consistent with previous findings, where the kinetic constant *b* depended exclusively on the substrate concentration [30]. In the present study, the protein concentration was constant because the same milk formulation was used in all experiments. This explains the poor correlation of the kinetic constant *b* with the T and E. Regression analysis and the analysis of variance, shown in Table 3, Table 4 and Table 5. The response surfaces from the reduced models for the kinetic constant *a* were plotted against T and E in Figure 2.

**Figure 3:**
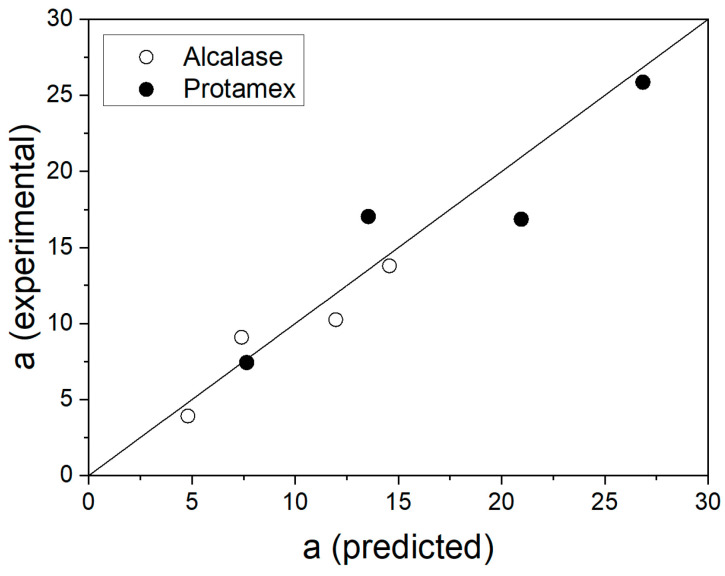
Correlation between predicted and experimental values of the kinetic constant *a* for the hydrolysis curves obtained in validation experiments with Alcalase (R^2^ = 0.9830) and Protamex (R^2^ = 0.9295). The correlation for both data is R^2^ = 0.9528.

**Figure 4:**
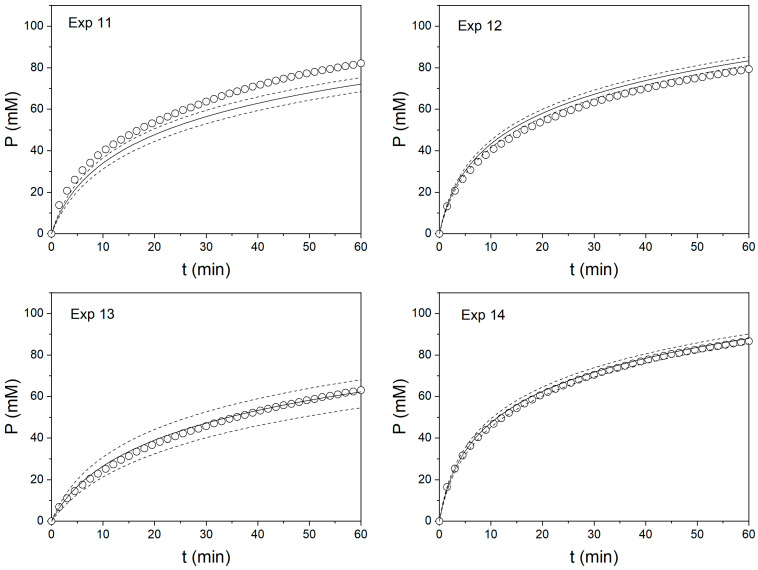
Predicted and experimental hydrolysis curves for the hydrolysis of milk proteins with Alcalase. Experimental data (circles), predicted curves (continuous line) and confidence interval of predicted curves at 95% probability (dashed lines) for experiments presented in Table 6.

**Figure 5:**
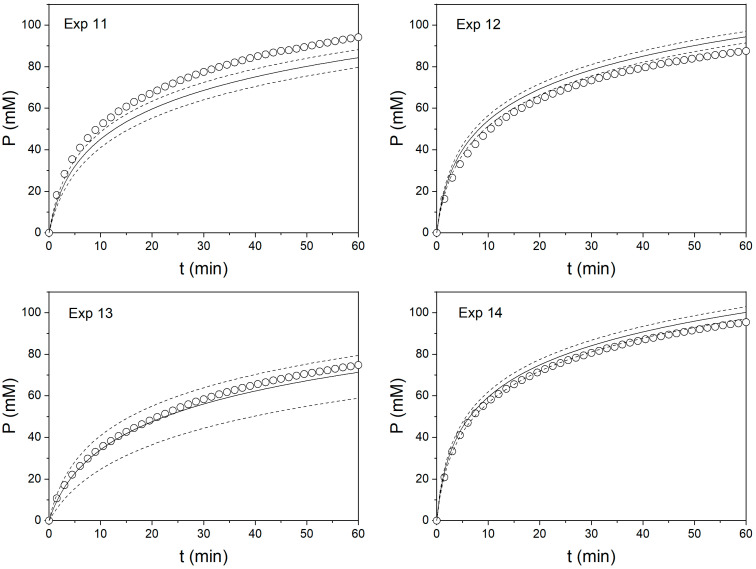
Predicted and experimental hydrolysis curves for the hydrolysis of milk proteins with Protamex. Experimental data (circles), predicted curve (continuous line) and confidence interval of predicted curves at 95% probability (dashed lines) for experiments presented in Table 6.

The ANOVA results shown in Table 3, Table 4 and Table 5 indicated that these models were significant in describing the kinetic constant *a* as a function of operating conditions, with the exception of the kinetic constant *a* for Neutrase (*p* > 0.01). This result was explained by the thermal inactivation of Neutrase. A better fitting can be achieved at lower temperatures for Neutrase. Considering the results obtained with Alcalase and Protamex, the polynomial models can be used to calculate the values of the kinetic constant *a* from a set of operating conditions (T, E) and plot the hydrolysis curves considering the fixed values for the kinetic constant *b*. The predictability of these models was tested with additional experiments, which are presented in Table 6. The models for kinetic constant *a* were used to calculate their predicted values, while the experimental values were calculated from the fitting of the hydrolysis curves from validation experiments. The predicted vales of the kinetic constant *a* were correlated against their experimental values in Figure 3. Good agreement was obtained for both Alcalase (R^2^ = 0.9830) and Protamex (R^2^ = 0.9295) independently and with both proteases together (R^2^ = 0.9528). For Neutrase, high prediction errors were obtained, thus, this protease was excluded from validation experiments. This error is explained by significant thermal inactivation of Neutrase. The predicted hydrolysis curves are plotted in Figure 4 and Figure 5 for Alcalase and Protamex, respectively.

The predicted hydrolysis curves were in good agreement with the experimental curves, despite the errors observed in Table 6 for the kinetic constant *a*, reaching approximately 20% in some cases. Indeed, the predicted DH after 60 min of reaction resulted in less than 5% error for most of the cases (Table 7). This result is an important source of validation for the present model because it can not only be used to predict the DH, but also to study the reactor performance and design to achieve a certain DH value. As noted in a previous article [30], the prediction quality of this model can still be improved by using a different experimental design and/or expanding the studied range of the operating conditions. Furthermore, this methodology allowed us to compare the performance of three different commercial proteases. It was observed that in the range of temperatures studied, Neutrase was rapidly inactivated, achieving a lower DH after 60 min of reaction compared to Alcalase and Protamex. Protamex showed an initial rate twice that observed for Alcalase. This performance is reflected in the values of the kinetic constant *a* (Table 2). Nevertheless, Alcalase reached almost the same values of DH as Protamex after 60 min of reaction. This performance is reflected in the lower values of the kinetic constant *b* obtained with Alcalase (Table 2). The performance of Protamex can be explained by a possible higher thermal inactivation and/or product inhibition than Alcalase. In resolution, this methodology can be used to evaluate the performance of any protease under different operating conditions. Different milk sources or formats can be evaluated using this methodology. Considerations about the nitrogen content must be prevented for the proper calculation of the DH. In the case that autotitration is not available, a different analytical technique can be used for the quantification of released α-NH, such as the TNBS [34] or OPA [31] methods. Sample withdrawal is required in these methods in order for the sample to be mixed with the proper reagent. If parameter conditions other than protease dose needs to be tested, such as milk concentration, pH or E/S, a new factor can be added to the polynomial equation after including this parameter in the experimental design. Moreover, different experimental designs can be tried if better statistical properties are achieved in the experimental configuration. Thus, the actual methodology offers not only reliability, but also versatility.

The logarithmic equation has largely been demonstrated to be a reliable model because of its good description, explanation, and prediction. The proposed modeling tool will improve the study of the correlation between DH and allergenicity during the enzymatic hydrolysis of milk proteins. An intermediate correlation between DH and allergenicity is further needed. In this way, DH can be used as an objective and quality control parameter during the hydrolysis process, thus allowing the reaction to be stopped at the desired allergenicity. This improvement will allow us to establish the operating conditions to produce a specific DH in the milk protein hydrolysate and, consequently, a specific functional property, for example, a specific allergenicity reduction.

## 4. Conclusions

The performance of the enzymatic hydrolysis of milk proteins was characterized and mathematically modeled against the temperature and protease concentration. The enzymatic hydrolysis performance was readily and accurately predicted using this modeling methodology to compare the protease efficiency and temperature effect. The temperature and protease concentration significantly affected the initial rate of hydrolysis, while the reaction extension was not affected. The model allows the possibility of studying the effect of additional variables, such as operating conditions, by simply adding variables to the polynomial equation and using an adequate experimental design. This methodology can be used in future research to test the effect of hydrolysis conditions on the reduction in antigenicity of hydrolysates. In this way, antigenicity can be correlated with operating conditions and the process can be designed to obtain hypoallergenic milk products.

## Figures and Tables

**Table 1:** Codified values for operating conditions of temperature and protease concentration.

Variable	Levels				
	−1.4	−1.0	0	+1.0	+1.4
E (mUA)	19	50	125	200	231
T (°C)	48	50	55	60	62

**Table 2:** Experimental CCC design and kinetic constant values obtained from fitting to hydrolysis curves and %DH after 60 min of reaction.

Exp	Variables			Alcalase			Neutrase			Protamex		
	*x*_1_ (S)	*x*_2_ (E)	*x*_3_ (T)	a ± se (mM/min)	b ± se (mM^−1^)	R^2^	a ± se (mM/min)	b ± se (mM^−1^)	R^2^	a ± se (mM/min)	b ± se (mM^−1^)	R^2^
1	−1.4	0.0	−1.4	4.06 ± 0.015	0.0494 ± 1.36 × 10^−4^	0.9940	25.6 ± 0.137	0.135 ± 2.02 × 10^−4^	0.9967	9.15 ± 0.030	0.0631 ± 9.98 × 10^−5^	0.9966
2	−1.0	1.0	−1.0	7.56 ± 0.027	0.0432 ± 9.01 × 10^−5^	0.9955	30.2 ± 0.196	0.104 ± 1.93 × 10^−4^	0.9923	16.5± 0.042	0.0509 ± 5.38 × 10^−5^	0.9983
3	−1.0	−1.0	−1.0	2.89 ± 0.009	0.0498 ± 1.34 × 10^−4^	0.9955	8.76 ± 0.011	0.105 ± 5.28 × 10^−5^	0.9996	4.36 ± 0.010	0.0542 ± 8.37 × 10^−5^	0.9980
4	0.0	1.4	0.0	15.2 ± 0.038	0.0356 ± 4.39 × 10^−5^	0.9980	42.7 ± 0.122	0.080 ± 6.38 × 10^−5^	0.9985	26.6 ± 0.050	0.0409 ± 2.99 × 10^−5^	0.9991
5	0.0	0.0	0.0	9.46 ± 0.042	0.0359 ± 9.36 × 10^−5^	0.9928	26.3 ± 0.048	0.082 ± 4.68 × 10^−5^	0.9993	16.1 ± 0.030	0.0407 ± 3.44 × 10^−5^	0.9990
6	0.0	−1.4	0.0	2.15 ± 0.003	0.0508 ± 8.14 × 10^−5^	0.9987	4.96 ± 0.007	0.082 ± 6.66 × 10^−5^	0.9993	2.93 ± 0.006	0.0498 ± 8.65 × 10^−5^	0.9981
7	1.0	1.0	1.0	21.7 ± 0.042	0.0361 ± 2.96 × 10^−5^	0.9990	86.5 ± 0.593	0.078 ± 1.28 × 10^−4^	0.9933	40.7 ± 0.126	0.0386 ± 4.12 × 10^−5^	0.9979
8	1.0	−1.0	1.0	5.76 ± 0.015	0.0353 ± 6.53 × 10^−5^	0.9974	22.0 ± 0.124	0.085 ± 1.58 × 10^−4^	0.9934	9.44 ± 0.014	0.0392 ± 3.19 × 10^−5^	0.9993
9	1.4	0.0	1.4	17.8 ± 0.045	0.0343 ± 4.01 × 10^−5^	0.9981	105.8 ± 1.08	0.090 ± 2.04 × 10^−4^	0.9866	30.2 ± 0.113	0.0411 ± 5.69 × 10^−5^	0.9967
10	0.0	0.0	0.0	10.4 ± 0.035	0.0401 ± 7.31 × 10^−5^	0.9962	37.7 ± 0.091	0.084 ± 5.79 × 10^−5^	0.9989	16.5 ± 0.031	0.0399 ± 3.38 × 10^−5^	0.9990

**Table 3:** Regression and variance analysis for RSM models for kinetic constants *a* and *b* for the enzymatic hydrolysis of milk proteins with Alcalase.

Regression analysis for kinetic constant $a=9.69+4.56x_{1}+4.88x_{2}+2.82x_{1}x_{2}$					
Predictor	Coefficient	se	t_calc_	P	
*β*_0_	9.694	0.350	27.68	1.47 × 10^−7^	
*β*_1_	4.563	0.392	11.65	2.41 × 10^−5^	
*β*_2_	4.880	0.392	12.46	1.63 × 10^−5^	
*β*_12_	2.820	0.554	5.093	0.00223	
R^2^ = 0.9814		R^2^ aj = 0.9721			
Analysis of variance for kinetic constant *a*					
Source of variation	Degrees of freedom	Sum of squares	Mean square	F	P
Regression	4	388.8616	97.2154	66.04	0.000162
Error	5	7.3599	1.47198		
Total	9	396.2215			
Regression analysis for kinetic constant $b=0.0410-0.00537x_{1}-0.00344x_{2}$					
Predictor	Coefficient	se	t_calc_	P	
*β*_0_	0.04103	0.00109	37.75	2.38 × 10^−9^	
*β*_1_	−0.00537	0.00122	4.42	0.00310	
*β*_2_	−0.00344	0.00122	2.83	0.0253	
R^2^ = 0.7972		R^2^ aj = 0.7393			
Analysis of variance kinetic constant *b*					
Source of variation	Degrees of freedom	Sum of squares	Mean square	F	P
Regression	3	0.0003251	0.000108	7.864	0.01679
Error	6	0.0000827	0.000014		
Total	9	0.0004078			

**Table 4:** Regression and variance analysis for RSM models for kinetic constants *a* and *b* for the enzymatic hydrolysis of milk proteins with Neutrase.

Regression analysis for kinetic constant $a=25.07+22.86x_{1}+17.41x_{2}+17.49x_{1}^{2}$					
Predictor	Coefficient	se	t_calc_	P	
*β*_0_	25.07	6.53	3.84	0.00855	
*β*_1_	22.86	4.98	4.59	0.00375	
*β*_2_	17.41	4.98	3.49	0.01291	
*β*_11_	17.49	5.96	2.94	0.02610	
R^2^ = 0.8746		R^2^ aj = 0.8119			
Analysis of variance for kinetic constant *a*					
Source of variation	Degrees of freedom	Sum of squares	Mean square	F	P
Regression	4	8322.2	2080.55	8.72	0.0177
Error	5	1193.2	238.63		
Total	9	9515.4			
Regression analysis for kinetic constant $b=0.0809-0.0135x_{1}+0.0147x_{1}^{2}$					
Predictor	Coefficient	se	t_calc_	P	
*β*_0_	0.08091	0.00188	42.98	9.64 × 10^−10^	
*β*_1_	−0.01352	0.00144	9.40	3.21 × 10^−5^	
*β*_11_	0.01470	0.00172	8.56	5.92 × 10^−5^	
R^2^ = 0.9585		R^2^ aj = 0.9466			
Analysis of variance kinetic constant *b*					
Source of variation	Degrees of freedom	Sum of squares	Mean square	F	P
Regression	3	0.002673	0.000891	46.17	0.000154
Error	6	0.000116	0.000019		
Total	9	0.002788			

**Table 5:** Regression and variance analysis for RSM models for kinetic constants *a* and *b* for the enzymatic hydrolysis of milk proteins with Protamex.

Regression analysis for kinetic constant $a=17.25+7.40x_{1}+9.60x_{2}+4.79x_{1}x_{2}$					
Predictor	Coefficient	se	t_calc_	P	
*β*_0_	17.25	0.818	21.09	7.41 × 10^−7^	
*β*_1_	7.40	0.914	8.09	0.000191	
*β*_2_	9.60	0.914	10.50	4.38 × 10^−5^	
*β*_12_	4.79	1.293	3.71	0.0100	
R^2^ = 0.9693		R^2^ aj = 0.9540			
Analysis of variance kinetic constant *a*					
Source of variation	Degrees of freedom	Sum of squares	Mean square	F	P
Regression	4	1267.3	316.83	39.47	0.000565
Error	5	40.1	8.03		
Total	9	1307.4			
Regression analysis for kinetic constant $b=0.0423-0.00731x_{1}+0.00436x_{1}^{2}$					
Predictor	Coefficient	se	t_calc_	P	
*β*_0_	0.0423	0.00157	27.01	2.44 × 10^−8^	
*β*_1_	−0.00731	0.00120	6.10	0.000489	
*β*_11_	0.00436	0.00143	3.05	0.0186	
R^2^ = 0.8693		R^2^ aj = 0.8320			
Analysis of variance kinetic constant *b*					
Source of variation	Degrees of freedom	Sum of squares	Mean square	F	P
Regression	3	0.000534	0.000178	13.30	0.004637
Error	6	0.000080	0.000013		
Total	9	0.000614			

**Table 6:** Predicted and experimental values for kinetic constants *a* and *b* obtained from validation experiments with Alcalase and Protamex.

Exp	Variables		Alcalase			Protamex		
	*x*_1_ (T)	*x*_2_ (E)	*a* ± se			*a* ± se		
			Predicted	Experimental	Error (%)	Predicted	Experimental	Error (%)
11	−0.5	0	7.41 ± 0.44	9.10 ± 0.02	18.6	13.6 ± 2.51	17.0 ± 0.01	20.4
12	0.5	0	12.0 ± 0.44	10.2 ± 0.01	16.8	21.0 ± 2.51	16.9 ± 0.03	24.2
13	0	−1	4.81 ± 0.58	3.92 ± 0.01	22.8	7.65 ± 3.29	7.44 ± 0.01	2.9
14	0	1	14.6 ± 0.58	13.8 ± 0.02	5.7	26.8 ± 3.29	25.9 ± 0.03	3.8

**Table 7:** Predicted and experimental values for DH after 60 min of milk protein hydrolysis from validation experiments with Alcalase and Protamex.

Exp	Variables		DH					
	*x*_1_ (T)	*x*_2_ (E)	Alcalase			Protamex		
			Predicted	Experimental	Error (%)	Predicted	Experimental	Error (%)
11	−0.5	0	21.8	25.7	15.3	25.5	29.6	14.0
12	0.5	0	25.2	24.8	1.5	28.5	27.5	3.8
13	0	−1	18.8	19.6	3.9	21.6	23.4	7.9
14	0	1	26.6	27.2	2.2	30.3	30.1	0.6

## Data Availability

Data is contained within the article.
